# Seasonal and Diurnal Variation in Leaf Phenolics of Three Medicinal Mediterranean Wild Species: What Is the Best Harvesting Moment to Obtain the Richest and the Most Antioxidant Extracts?

**DOI:** 10.3390/molecules25040956

**Published:** 2020-02-20

**Authors:** Antonella Gori, Luana Beatriz Nascimento, Francesco Ferrini, Mauro Centritto, Cecilia Brunetti

**Affiliations:** 1 University of Florence, Department of Agriculture, Food, Environment and Forest (DAGRI), Section Woody Plants, 50019 Sesto Fiorentino (Florence), Italy; antonella.gori@unifi.it (A.G.); luananascimento@ufjr.br (L.B.N.); francesco.ferrini@unifi.it (F.F.); 2 National Research Council of Italy, Institute for Sustainable Plant Protection (IPSP), 50019 Sesto Fiorentino (Florence), Italy; mauro.centritto@cnr.it

**Keywords:** antioxidant capacity, *Cistus incanus*, DPPH, environmental factors, flavonoids, *Phyllirea latifolia*, *Pistacia lentiscus*, polyphenols, seasonality, tannins

## Abstract

Mediterranean plants biosynthesize high amounts of polyphenols, which are important health-promoting compounds. Leaf polyphenolic composition changes according to environmental conditions. Therefore, it is crucial to know the temporal variation in their production. This study aimed to: i) evaluate the monthly and daily changes in polyphenols of *Phyllirea latifolia*, *Cistus incanus*, and *Pistacia lentiscus* to identify their best harvesting moment, ii) verify the possible correlations between phenolic production and temperature and irradiation, iii) evaluate their antioxidant capacity using 2,2-diphenyl-1-picrylhydrazyl (DPPH) and hydroxyl radical (OH)scavenging assays. The extracts of leaves harvested at 8:00, 13:00 and 18:00, in May, July, and October for two years were analysed by HPLC-DAD. Both “month” and “time of the day” affected the polyphenolic content in all species. July at 13:00 was the best harvesting moment for all polyphenolic classes of *P. latifolia* and only for some classes of *C. incanus* and *P. lentiscus*. Environmental parameters positively correlated with the polyphenols of *C. incanus* and *P. latifolia*, while the antioxidant capacity only varied in this last species, reaching the highest value in July. Results of the study allow to determine the balsamic time for each species. Moreover, the relationship between polyphenols and environmental data can be useful for the cultivation of these plants under controlled conditions.

## 1. Introduction

The Mediterranean basin is one of the richest places in plant biodiversity [1]. This region is dominated by arid and semi-arid ecosystems, in which extended dry periods over the summer are combined with high air temperatures [2]. Mediterranean species have evolved several physiological and biochemical adaptative traits, including the capacity to synthesize large amounts of secondary metabolites, to cope with such harsh environmental conditions [3,4,5,6]. In particular, polyphenols have an impressive multiplicity of protective roles for the plants, taking part in the defense against pathogens, drought, oxidative stress, and excess of light/UV radiation [7,8].

Besides their physiological and ecological functions, polyphenols possess many health-promoting benefits, including protection against cardiovascular diseases, cancer, diabetes, and neurological disorders, and have been utilized by humans as healing agents for centuries [7,9,10,11,12]. 

In the last few years, there has been a renewed interest in phytotherapic products, and many Mediterranean phenolic-rich species may represent unrivalled sources of nutraceutical, cosmetic, and pharmacological products [3,4,5]. This revival has triggered interest in plant polyphenols, methods for their extraction, and the bioactivity of these compounds [13,14,15,16].

Until nowadays, medicinal plant material has been mainly harvested from nature [17]. Cultivation of Mediterranean phenolic-rich species would be a sustainable alternative to wild crafting, also considering that many of these plants, thanks to their high resistance to both biotic and abiotic stresses, can grow in poor and marginal lands, with very low cultivation inputs (e.g. no use of irrigation, fertilizers, pesticides, etc.) [18,19]. In addition, controlled production of Mediterranean medicinal plants could be a key factor to reach not only the standardization, but also the quality and safety required by the regulatory authorities [20].

To fully utilize these plants as sources of natural products, it is essential not only to characterize polyphenols in their leaves, but to also check the variation in their production to obtain enriched extracts with, consequently, pharmacological and/or economical values [17,21]. Indeed, the amount of polyphenols changes according to phenological and developmental stages of the plants and to different environmental conditions [5,15,22]. Regarding this issue, studies concerning the seasonal and diurnal polyphenolic changes are still scarce [23], especially for Mediterranean plants, possibly due to the difficult and the laborious nature of such work.

*Phillyrea latifolia* L. (Oleaceae), *Cistus incanus* L. (Cistaceae) (syn. *Cistus creticus*, *Cistus × incanus*) and *Pistacia lentiscus* and L. (Anacardiaceae) are among the most widespread species in the Mediterranean basin and are commonly used in the folk medicine of this area [23,24,25,26].

Recently, different biological activities have been demonstrated for the leaf extracts of these species and they have furnished scientific evidence to their traditional use. *Cistus incanus* is the main component of the medicinal product CYSTUS052® (Dr. Pandalis Urheimische Medizin GmbH und Co. KG, Germany), which has given promising results as anti-HIV agent [27] and in the treatment of infections of the upper respiratory tract [28]. Furthermore, Kutcha et al. [29] have demonstrated that a regular intake of *C. incanus* infusion has a positive impact on lipid metabolism, while its powder can be utilized as an additional ingredient during the bread-making process to obtain fortified bread with high nutritive and antioxidant capacity [30]. In addition to the well-known health-promoting effects of the resin (mastic gum) extracted from *P. lentiscus* and its food applications [31,32], it has recently been shown that leaves of this plant are a rich source of antioxidant polyphenols, in particular tannins [33]. Tannin-rich extracts of *P. lentiscus* leaves have exhibited anthelmintic activity that could be applied in the infection of grazing ruminants with gastro-intestinal nematodes [34], as well as significant antimicrobial activity, which can be utilized to reduce microbial spoilage during storage of fresh foods [35]. Long-term treatments with *P. lentiscus* extracts significantly reduce the cognitive decline in mice induced by chronic exposure to aluminum chloride [36], thus supporting their potential role in the prevention of neurodegenerative disorders [37]. In addition, non-boiled aqueous extracts of *P. lentiscus* have shown promising *in vivo* hepatoprotective activity by reducing the liver hepato-specific enzymes in the serum and can be applied for the treatment of hepatic jaundice [38]. This effect was also obtained with the boiled extracts of *P. latifolia* leaves, which resemble the traditional way of decoction preparations used as medicinal tea for weight loss and hyperglycaemia [39].

Despite the knowledge about the polyphenolic composition of the extracts of these species [6,23,24,40,41,42,43,44], qualitative and quantitative data on their seasonal and diurnal variation, as well as the relationship with environmental parameters have not been reported yet. 

In this context, this study aimed to:

(i) evaluate the monthly and daily changes of polyphenols in leaves of *P. latifolia*, *C. incanus*, and *P. lentiscus* collected in their natural environment in order to identify the best harvesting moment (balsamic time) for each species;
(ii) evaluate the possible correlation between phenolic content and temperature and irradiation to know how these environmental factors modulate leaf phenolic production; and,
(iii) verify the monthly variations in antioxidant capacity of the richest-polyphenol extracts.

## 2. Results and Discussion

### 2.1. General Phenolic Profile of the Leaf Extracts 

**Figure 1:**
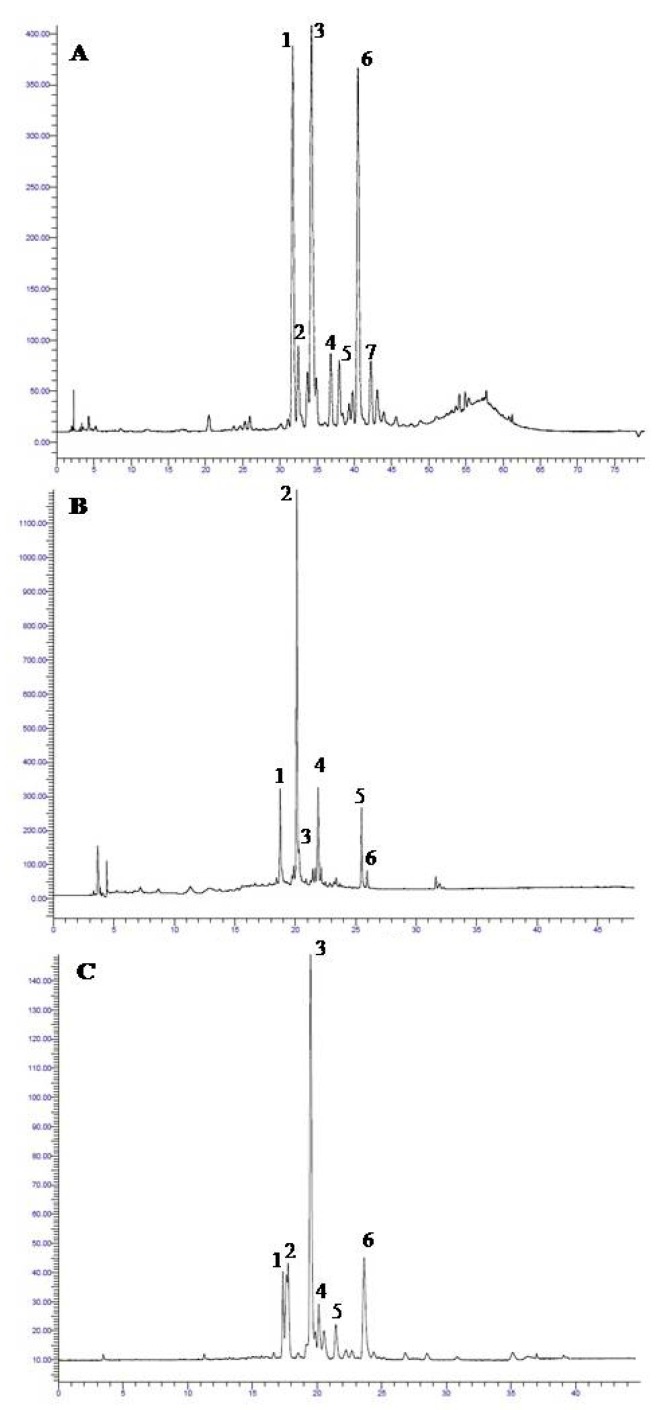
Chromatograms (350 nm) of leaf ethanolic extracts of *Phillyrea latifolia* (**A**), *Cistus incanus* (**B**) and *Pistacia lentiscus* (**C**). In *P. latifolia* (**A**), rutin (1), luteolin-7-O-rutinoside (2), luteolin-7-O-glucoside (3),kaempferol derivative (4), caffeic acid derivative (5), luteolin-4’-O-glucoside (6) and luteolin-7-O-derivative (7) were the major compounds. In *C. incanus* (**B**) myricetin-3-O-glucoside (1), myricitrin (2), quercetin-3-O-glucoside (3), rutin (4), quercetin-3-O-pentoside (5), and quercitrin (6) were the most abundant flavonoids. In *P. lentiscus* (**C**), myricetin-3-O-galactoside (1), myricetin-3-O-rutinoside (2), myricitrin (3), myricetin derivative (4), quercetin-3-O-arabinoside (5), and quercitrin (6) were detected.

Different polyphenols were detected and quantified in leaf ethanolic extracts of *P. latifolia* (Figure 1A, Table S1), *C. incanus* (Figure 1B, Table S2) and *P. lentiscus* (Figure 1C, Table S3). Flavonols (quercetin and kaempferol) and flavones (apigenin and luteolin) derivatives (Figure 1A), as well as derivatives of caffeic acid were detected in *P. latifolia* (Figure 1A, Table S1). *C. incanus* mainly showed flavonols (quercetin, kaempferol, and myricetin derivatives) (Figure 1B) and tannins (Table S2). This phenolic profile was similar to that of *P. lentiscus* (Figure 1C, Table S3).

**Figure 2:**
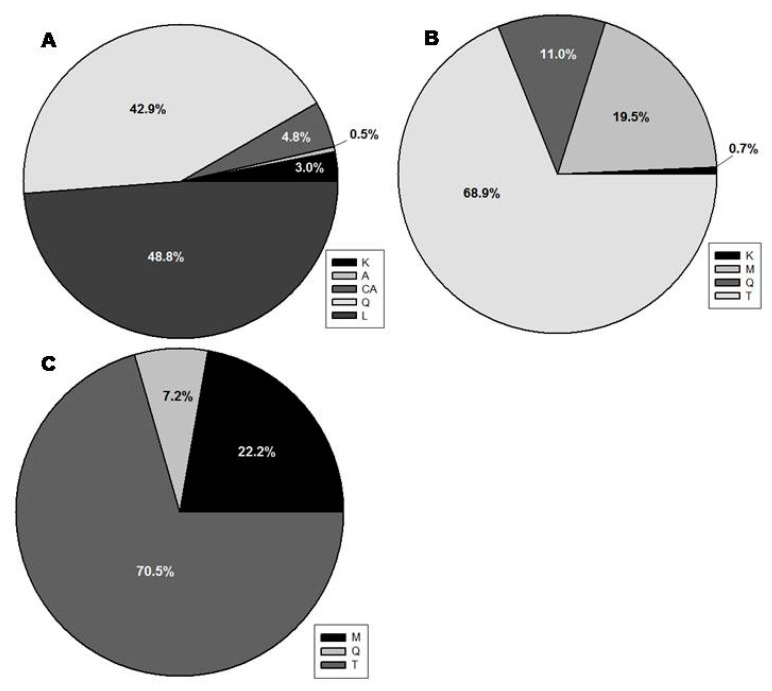
Percentage of each phenolic class (K—kaempferol derivatives; A—apigenin derivatives; CA—caffeic acid derivatives; L—luteolin derivatives; M—myricetin derivatives; Q—quercetin derivatives; T—tannin derivatives) relative to the total phenolic content (TPC—100%) in extracts of *Phillyrea latifolia* (**A**), *Cistus incanus* (**B**), and *Pistacia lentiscus* (**C**) leaves. For *P. latifolia*, L and Q are the most abundant classes of phenolics, corresponding to around 92% of TPC. Flavonoids (L, Q, K and A) represents around 95% of the phenolic compounds in the extracts. For *C. incanus* (**B**) and *P. lentiscus* (**C**), tannins (T) represents around 70% of the TPC, and all the other compounds are flavonoids (K, Q, and M for *C. incanus*, and Q and M for *P. lentiscus*), with M being the major flavonoid class in leaf extracts of both species.

All three species showed to be rich in flavonoids. These compounds receive special attention nowadays for their biological activities, such as antimicrobial, anti-inflammatory, and anticancer [9,12]. Given the high flavonoid content of the studied species (Figure 2), they would represent raw materials for potential different products, including pharmaceutics and nutraceuticals that are based both on their traditional uses [23,24,25,26] and the scientific evidence [28,29,37,39].

Regarding the different classes of polyphenols, in *P. latifolia* (Figure 1A), rutin (1), luteolin derivatives (peaks 2, 3, 5, 6, 7), and a kaempferol derivative (4) were the major flavonoids detected. A derivative of caffeic acid (5) was also detected. Indeed, while considering the percentage amount of each phenolic class (Figure 2), luteolin (L) and quercetin (Q) derivatives were the major polyphenols in *P. latifolia*, representing around 43% and 49% of the total phenolic content (TPC), respectively. Together, these compounds summed more than 90% of TPC (Figure 2A). Flavonoids with quercetin and luteolin aglycones were already described as important constituents of *P. latifolia* leaves [23,34,39,40]. Both aglycones have a catechol group in B ring of the flavonoid structure, a feature that confers them a high potential as free-radical scavengers [45]. 

For *C. incanus* (Figure 1B), myricetin derivatives such as myricetin-3-O-glucoside (1) and myricitrin (2), as well as quercetin derivatives, including quercetin-3-O-glucoside (3), rutin (4), quercetin-3-O-pentoside (5), and quercitrin (6), were the most abundant flavonoids. Myricetin and quercetin glycosides were also the most important flavonoids that were detected in *P. lentiscus* leaves (Figure 1C). For both *C. incanus* (Figure 2B) and *P. lentiscus* (Figure 2C), tannin derivatives (T) represented around 70% of the TPC.

Similar leaf phenolic composition was already described in literature for both species, and these compounds are thought to be main responsible for their biological activities [27,33,44,46,47]. 

### 2.2. Seasonal and Diurnal Variation in Polyphenolic Composition

**Figure 3:**
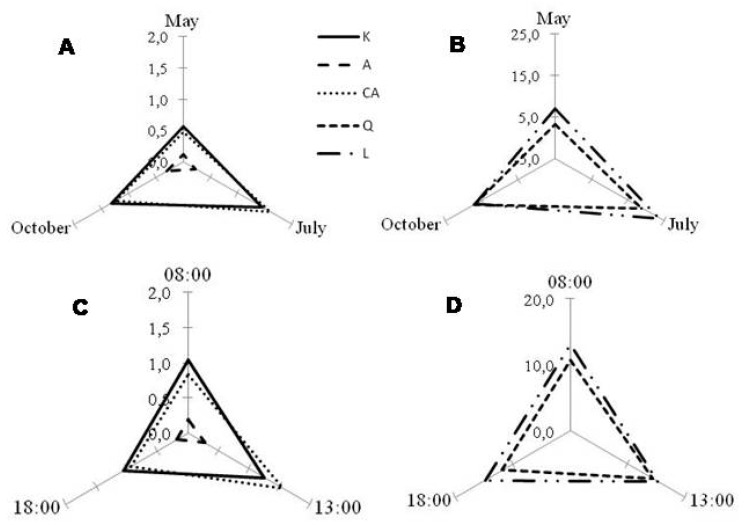
Monthly (**A** and **B**) and diurnal (**C** and **D**) variation of the different polyphenolic classes detected in *Phillyrea latifolia* leaf ethanolic extracts: A—apigenin derivatives; CA—caffeic acid derivatives; K—kaempferol derivatives; L—luteolin derivatives, and Q—quercetin derivatives. In general, for all compounds, monthly variation (**A** and **B**) was more prominent than diurnal (**C** and **D**), with May as the worst month for harvesting, regardless the time of the day. For caffeic acid derivatives (CA), irrespective of month, harvesting at 13:00 is better than other day times (**C**). Graphics were obtained with the mean values of the consecutive years of analysis (2014 and 2015, n = 18). Compounds were represented in the same graphic according to their amounts.

**Figure 4:**
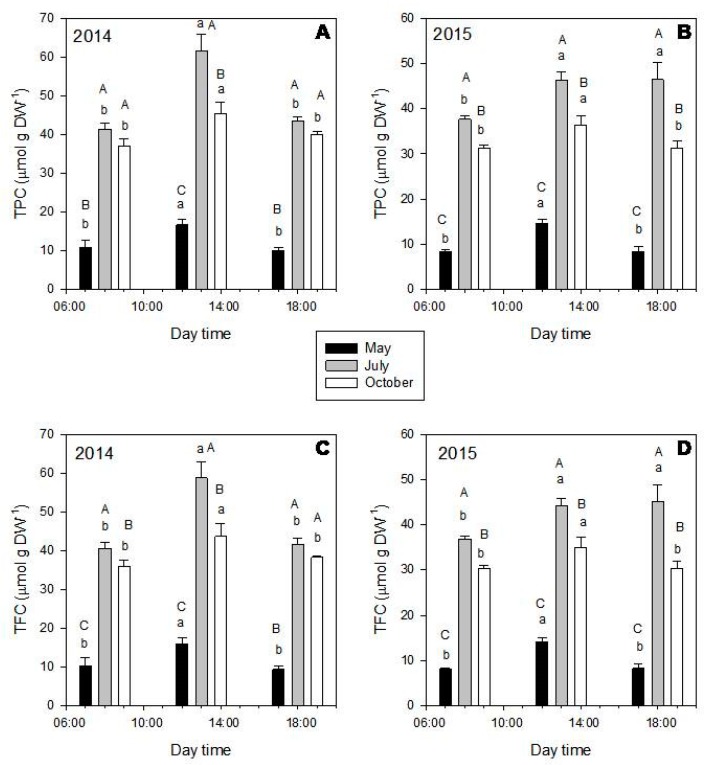
Total phenolic (TPC—upper graphics) and total flavonoid contents (TFC—down graphics) of *Phillyrea latifolia* extracts of leaves harvested in 2014 (left) and 2015 (right), during the different months (May—black bars; July—gray bars; October—white bars) and day times (8:00; 13:00, and 18:00). Mean values ± SD in μmol gDW^−1^ (n = 3). Data was analyzed by two-way ANOVA test, with Tukey post-test, after variance homogeneity analysis by Levene’s test and normality analysis by Shapiro-–Wilk test. Equal capital letters indicate no statistical differences between the monthly results (at the same time) and lowercase letters indicate no statistical differences between daily results (inside the same month) (*p* ≤ 0.05).

Different seasonal and diurnal behaviors in phenolic content were observed for all the species studied. For *P. latifolia*, both month and time of the day significantly affected the content of polyphenols (*p* < 0.001, Table S1, Figure 3 and Figure 4). However, monthly variations were more pronounced than diurnal ones (Figure 3). Particularly, leaves collected in July (summer) showed higher polyphenolic contents, especially when compared to May (spring) and October (autumn) (Table S1, Figure 3A,B). On a daily level, caffeic acid derivatives showed higher variation compared to other polyphenolic classes (Figure 3C). Indeed, for this class of phenolics, the highest concentrations were observed during the central hours of the day, irrespective of the month considered. This increase from morning to midday might likely be related to the changes in diurnal solar irradiance [48] thanks to their protective role against UV wavelengths and their peculiar location in the epidermis (glandular trichomes) of *P. latifolia* leaves [49,50].

In general, both TPC (Figure 4, upper graphics) and TFC (Figure 4, down graphics) were significantly affected by the month and time of the day (*p* < 0.001). Particularly, TPC and TFC showed higher values in July (gray bars), followed by October (white bars) and May (black bars) (Figure 4). While considering the diurnal variations, TPC and TFC increased from 8:00 to 13:00 and decreased from 13:00 to 18:00. These diurnal behaviors agreed with the dynamics of the individual phenolic amounts (Table S1, Figure 3C,D), with higher values being obtained in leaves harvested in July at 13:00 (Table S1), except for apigenin derivatives, showing highest values at 13:00 in October (Table S1, Figure 3).

Data of our study support the view that polyphenols, especially phenylpropanoids, in addition to absorbing the most energetic solar wavelengths and act as UV filters, may counter the oxidative stress [51,52,53] generated by long exposure to the harsh environmental conditions of the Mediterranean summer days, characterized by high temperatures, high light intensity and concomitant drought [6].

**Figure 5:**
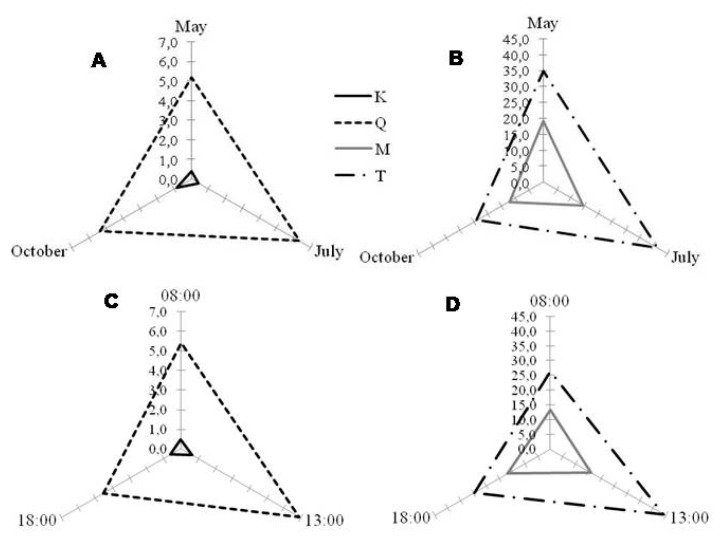
Monthly (**A** and **B**) and diurnal (**C** and **D**) variation of the different polyphenolic classes detected in *Cistus incanus* leaf ethanolic extracts: K—kaempferol derivatives; M—myricetin derivatives; Q—quercetin derivatives, and T—tannin derivatives. Regarding the month (**A** and **B**), for T and Q derivatives July showed to be the best; for K derivatives October was better and for M derivatives, it was May. Concerning diurnal variation (**C** and **D**), to reach higher amounts of Q, K, and T derivatives, harvesting at 13:00 is better, while for M derivatives, is 18:00. Graphics were obtained with the mean values of the consecutive years of analysis (2014 and 2015, *n* = 18). Compounds were represented in the same graphic according to their amounts.

**Figure 6:**
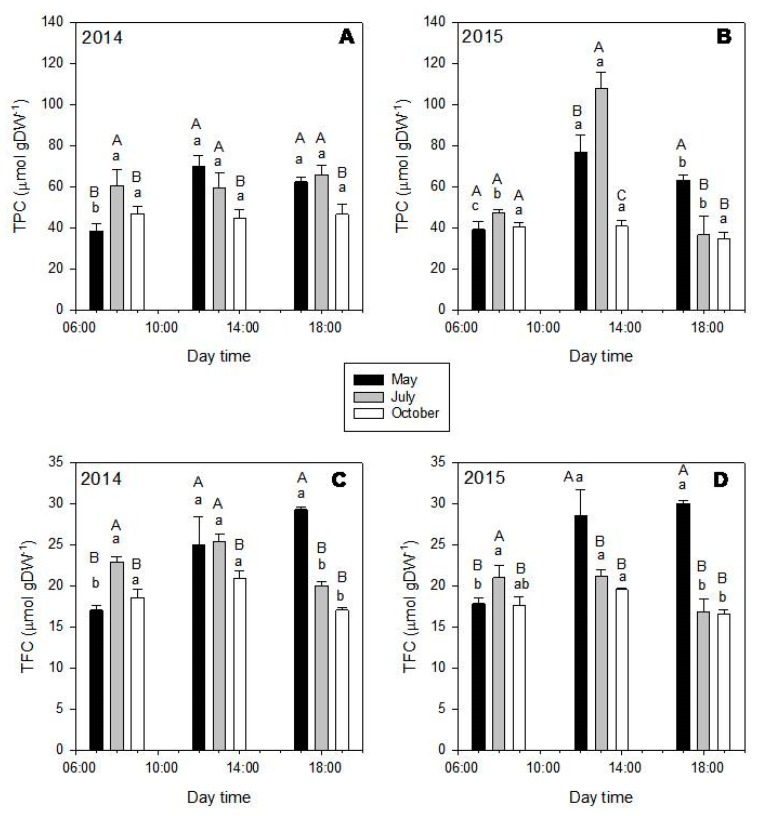
Total phenolic (TPC—upper graphics) and total flavonoid contents (TFC—down graphics) of *Cistus incanus* extracts of leaves harvested in 2014 (left) and 2015 (right), during the different months (May—black bars; July—grey bars; October—white bars) and day times (8:00; 13:00 and 18:00). In general, the highest values were obtained in May and July. Mean values ± SD in μmol gDW^−1^ (*n* = 3). Data was analyzed by two-way ANOVA test, with Tukey post-test, after variance homogeneity analysis by test and normality analysis by Shapiro–Wilk test. Equal capital letters indicate no statistical differences between the monthly results (at the same time) and lowercase letters indicate no statistical differences between daily results (inside the same month) (*p* ≤ 0.05).

Harvesting time and month had also a significant effect in leaf phenolics of *C. incanus* (*p* < 0.001, Table S2, Figure 5 and Figure 6). For this species, each class of polyphenols showed a different seasonal behavior, with the highest amounts of kaempferol derivatives in October (autumn), highest values of myricetin derivatives in May (spring), and of quercetin and tannin derivatives in July (summer) (Figure 5A B). On a daily timescale, the quercetin and tannin derivatives showed higher variation when compared to other classes, with the greatest values being obtained at 13:00 (Figure 5C,D; Table S2).

As previously mentioned, in *C. incanus*, tannins represent the main leaf polyphenolic fraction and they may play important ecological roles, such as preventing nitrogen depletion by their cycling chelating properties as well as protection against herbivory and pathogens [54,55,56,57]. These compounds, which were located in the trichomes [57], together with the noticeable ROS scavenging capacity of quercetin derivatives [45], could play a complementary role in the protection of *C. incanus* leaves against multiple environmental stresses that are generally exacerbated during the summer period.

In this species, TPC (Figure 6, upper graphics) and TFC (Figure 6, down graphics) also varied according to the month and time of the day (*p* < 0.001). In general, leaves that were harvested in May and July showed the highest values for both contents.

Similar results were also reported in literature for other *Cistus* species. For example, the seasonal analysis of total phenolics in *C. clusii* showed that higher amounts of these compounds were obtained in leaves collected in summer, followed by those harvested in spring and then in autumn. In addition, the authors showed a slightly difference between the behaviors of total phenols and the total flavonoids content [58]. 

**Figure 7:**
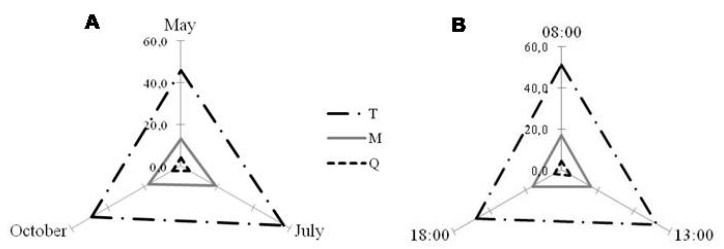
Monthly (**A**) and diurnal (**B**) variation of the different polyphenolic classes detected in *Pistacia lentiscus* leaf ethanolic extracts: M—myricetin derivatives; Q—quercetin derivatives, and T—tannin derivatives. For monthly variation (**A**), July seemed to be slightly better to reach higher amounts of T derivatives. While considering the time of the day (**B**), there is not a big difference between the content of the different compounds. Graphics were obtained with the mean values of the consecutive years of analysis (2014 and 2015, *n* = 18).

**Figure 8:**
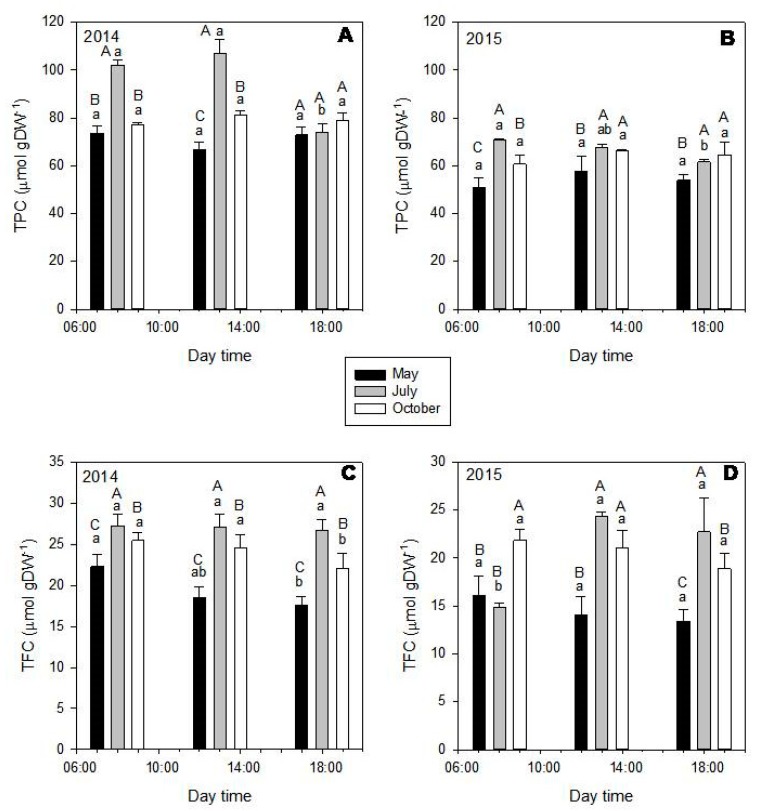
Total phenolic (TPC—upper graphics) and total flavonoid contents (TFC—down graphics) of *Pistacia lentiscus* extracts of leaves harvested in 2014 (left) and 2015 (right), during the different months (May—black bars; July—gray bars; October—white bars) and day times (8:00; 13:00; and, 18:00). In general, July was the month with the highest amount of TPC and TFC, followed by October and then May. Almost any variation related to time of the day was observed. Mean values ± SD in μmol gDW^−1^ (*n* = 3). Data was analyzed by two-way ANOVA test, with Tukey post-test, after variance homogeneity analysis by Levene’s test and normality analysis by Shapiro–Wilk test. Equal capital letters indicate no statistical differences between the monthly results (at the same time) and lowercase letters indicate no statistical differences between daily results (inside the same month) (*p* ≤ 0.05).

“Month” and “time of the day” had also a significant effect (*p* < 0.001) in polyphenols production in *P. lentiscus* (Table S3, Figure 7 and Figure 8). Leaves collected in July (summer) showed the highest amount of all polyphenol classes (T, M and Q), followed by October (autumn) and May (spring) (Table S3, Figure 7A). Considering the daily timescale, slighter changes were observed (Figure 7B). For Q, higher amounts were generally obtained at 13:00, similarly to what was observed in *C. incanus*. On the other hand, leaves collected at 13:00 and 18:00 in May and October had higher amounts of T (Table S3).

In *P. lentiscus*, gallotannins are reported to be located in the whole leaf tissues and may strength the cell walls, thus contributing to both leaf thickness and sclerophylly [6]. These anatomical features may improve protection against pathogen and insect attacks during blooming and fructification periods (Table 3) [55,56,57].

In *P. lentiscus*, TPF (Figure 8, upper graphics) and TFC (Figure 8, down graphics) were significantly affected by the sampling month (*p* < 0.001), while almost any variation in a daily timescale was observed. In general, leaves that were collected in July (summer) showed the highest total amount of polyphenols, followed by October (autumn) and then May (spring) (Figure 8; Table S3). Different from our findings, two other species of *Pistacia*, *P. chinensis*, and *P. atlantica*, showed higher total flavonoids and total phenolic contents in spring, when compared to summer and autumn [59,60].

The knowledge regarding diurnal and seasonal variability in polyphenol content is essential for determining the best harvesting moment for leaves of medicinal plants (balsamic time). The seasonal differential production of polyphenols is generally attributed to the distinct phenological stages (vegetative or reproductive) and also to the varied climatic conditions in each season, such as the length of the day, total irradiance, water availability, and temperature [6,15,61]. These both intrinsic and extrinsic factors can act direct in the expression of genes from polyphenols pathway, triggering their production to keep plant development and survival [51]. 

Although seasonal studies are common for different plant species, studies regarding diurnal effects on the production of polyphenols are scarce. In our study, the diurnal and seasonal production of polyphenols varied not only according to the species, but also to the classes of polyphenol considered. Indeed, each class, with its peculiar chemical structure and distribution in leaf tissues, has a specific role in plant metabolism [51].

### 2.3. Correlation with Environmental Parameters

Polyphenols play important roles in plant-environment interactions and the amount of these compounds can strongly change according to climate, allowing for plants to acclimate themselves to harsh conditions [22]. This is especially important for leaves, since these plant organs are probably the most plastic and responsive to abiotic factors [62].

For *P. latifolia*, temperature showed to be an important environmental factor for the production of different polyphenolic classes. A significant moderate positive influence of this factor in the content of caffeic acid, quercetin, and luteolin derivatives, as well as in TPC and TFC, was detected (Table 1). Thus, higher temperatures seemed to increase the production of these compounds. For *C. incanus*, temperature and solar irradiance showed a significant moderate positive influence on the content of quercetin and tannins derivatives. Besides, solar irradiance was shown to positively affect the TPC (Table 1). On the other hand, for *P. lentiscus*, no significant correlations were found between polyphenols and the environmental parameters considered (Table 1).

The relationship between polyphenols levels and environmental parameters can vary according to different species, and this can be attributed to the distinct taxonomic and ecological aspects of them [58]. A positive correlation between the increase of light intensity and UV-B radiation and the amount of phenolics has already been reported [63,64]. In addition, it has been previously shown that both high and low temperatures can positively affect the content of polyphenols [58,65,66,67]. 

It is important to highlight that *P. lentiscus* did neither show a diurnal variation in TPC and TFC (Figure 8) nor a variation in antioxidant capacity (Table 2). Therefore, this species, when compared to the other two, is less responsive to environmental changes. Moreover, since no correlation between polyphenols and temperature or irradiance were found for *P. lentiscus*, other environmental parameters (both biotic and abiotic) or biochemical changes associated to its phenological stages, could explain the seasonal variation in polyphenol composition observed for this species. Indeed, in the natural environment, abiotic and biotic factors can interact with each other, causing metabolic changes [68]. 

Our results suggest that abiotic factors, and in particular temperature for *P. latifolia* and temperature and irradiation for *C. incanus*, are important for modulating their leaf polyphenolic content. This could be an important information for further biotechnological studies of these plants, since these factors can be applied under controlled conditions to optimize the production of target compounds.

### 2.4. Antioxidant Capacity of the Extracts

We have evaluated the *in vitro* antioxidant potential of extracts of leaves collected each month at 13:00 in order to verify how seasonality can influence the antioxidant capacity of the extracts. Two different methods were applied: DPPH (2,2-diphenyl-1-picrylhydrazyl) and Hydroxyl Radical (OH)-Scavenging (HRS) assays, both based on a colorimetric reaction that can be spectrophotometrically monitored [69,70]. Results are given in EC_50_ values, which represent the effective concentration of a sample that is necessary for reaching 50% of the activity [71]. 

The EC_50_ values of the leaf extracts of the three different species are presented in Table 2. In the DPPH assay, *C. incanus* showed to be more effective as free-radical scavenger than *P. latifolia* and *P. lentiscus*, with values from two to 20 times lower than the others (Table 2).

In both DPPH and HRS assays, the obtained EC_50_ values were different according to the harvesting month only for *P. latifolia*. For this species, extracts of leaves collected in July showed the highest antioxidant capacity (lowest EC_50_; DPPH = 1.2 μg mL^−1^; HRS = 0.12 μg mL^−1^), followed by the extracts of leaves harvested in October (EC_50 DPPH_ = 2.2 μg mL^−1^; EC_50 HRS_ = 0.23 μg mL^−1^) and May (EC_50 DPPH_ = 9.6 μg mL^−1^; EC_50 HRS_ = 0.47 μg mL^−1^). These results are in accordance with the seasonal change in TPC of this species (Figure 4), which could be expected, since flavonoids, the most important polyphenolic class in *P. latifolia,* are strong antioxidants [45]. Therefore, an increase in the amount of these compounds in extracts should also enhance their antioxidant potential. 

For *C. incanus* and *P. lentiscus*, changes in TPC were not followed by a concomitant variation in the antioxidant capacity of their extracts in any of the tested methods (Table 2). The same was previously observed for leaf extracts of *Pistacia atlantica* [59]. 

Despite that a direct correlation between total polyphenol content and antioxidant activity is reported for many food and medicinal plants [72], the antioxidant activity of an extract can change according to its chemical composition. A leaf extract is a complex mixture of substances and, as such, its phenolic profile, more than the total amount of phenolics, may confer a different scavenger activity to the extract [14,72]. Moreover, some other non-phenolic antioxidants, including ascorbic acid, tocopherol, and carotenoids can be found in the leaves of these species [73], none of them evaluated in this study. 

## 3. Material and Methods

### 3.1. Plant Material, Study Area and Meteorological Data

Three plants of *Pistacia lentiscus* L., *Phillyrea latifolia* L., and *Cistus incanus* L. were randomly chosen from their natural habitat in the coastal dunes of Southern Tuscany, Italy (42°46’ N, 10°53’ E).

During two consecutive years (2014 and 2015), the leaves from branches at the top of the canopy were sampled in three hours of the day (daily analysis): 8:00 h, 13:00 h, and 18:00 h; in three different months: May (spring), July (summer), and October (autumn). Table 3 presents details of the phenological stage of each species, air temperature, precipitation and global irradiance in harvesting months at the site of collection of both years. The meteorological data were obtained by the weather station “Ponti di Badia”, located 7 km from the harvesting place.

### 3.2. Sampling and Biochemical Analyses

After collection, the leaves were immediately frozen in liquid nitrogen, kept at −80 °C, and then lyophilized. The material (150 mg) was extracted with 3 × 5.0 ml ethanol 75% (pH 2.5 adjusted with formic acid) and the supernatant partitioned with 3 × 5 mL of *n*-hexane. The ethanolic extract was then reduced to dryness, and the residue was resuspended with 1.0 mL of methanol/water solution (9:1 *v/v*) for the HPLC-DAD analysis.

The aliquots of the samples were injected into the Perkin^®^ Elmer Flexar liquid chromatograph that was equipped with a quaternary 200Q/410 pump and an LC 200 diode array detector (DAD) (all from Perkin Elmer^®^, Bradford^®^, CT, USA). The stationary phase consisted in a Zorbax^®^ SB-18 column (250 × 4.6 mm, 5 µm) (Agilent, Santa Clara, CA, USA), and kept at 30 °C. The eluents were (A) acidified water/acetonitrile (90/10, at pH 2.5 adjusted with HCOOH) and (B) acetonitrile/water (90/10, at pH 2.5 adjusted with HCOOH), for the analysis of the extracts of the three species. For *P. lentiscus* and *C. incanus*, the same following solvent gradient (*v/v*) was applied: 0–6 min. (0% B), 6–38 min. (0–20% B), 38–43 min. (20–100% B), 43–46 min. (100% B), and 46–48 min. (100–0% B). The flow elution was 0.8 mL min^−1^ with 10 μL of each sample injection. For *P. latifolia*, the gradient was as following: 0–8 min. (0% B), 8–38 min. (0–15% B), 38–43 min. (15% B), 43–51 min. (15–25% B), 51–59 min. (25–45% B), 59–67 min. (45–70% B), 67–72 min. (70–100% B), 72–77 min. (100% B), and 77–79 min. (100–0% B). The flow elution was 1.0 mL min^−1^ with 20 μL of each sample injection.

The extracts were analyzed in triplicate, within a wavelength range from 180 to 900 nm, and the chromatograms were obtained at 280, 330, and 350 nm. The identification and quantification of the polyphenols was carried out based on the retention time, UV spectral characteristics, and comparison with standards, as well as based on literature data. Different standards (caffeic and gallic acids; apigenin, kaempferol, luteolin, myricetin, and quercetin glycosides; and, epicatechin) were used to obtain calibration curves. 

The quantitative results of polyphenol (reported as μmol per g of dry weight, DW) were expressed in content of different classes of compounds: caffeic acid derivatives (CA), apigenin derivatives (A), kaempferol derivatives (K), luteolin derivatives (L), myricetin derivatives (M), quercetin derivatives (Q), and tannins derivatives (T). Each of them represents a sum of the concentration of singular compounds being detected in the extracts that belong to these classes (according to their individual identification). Besides, the total flavonoid content (TFC) and the total phenolic content (TPC) were also calculated.

### 3.3. Antioxidant Capacity Assays

The polyphenolic extracts showing highest amount of phenolics of each harvesting month (May, July, and October, from both years) were evaluated for free radical scavenging potential (antioxidant capacity) while using two methods: the DPPH (2,2-diphenyl-1-picrylhydrazyl) assay and the Hydroxyl Radical (OH)-Scavenging (HRS) assay. For DPPH assay [74], in 96-well microplates, 20 μL of a DPPH methanolic solution (150 μM) was added to 180 μL of each diluted sample or methanol (negative control). After 40 min. in the dark, the absorbance was detected at 518 nm while using a SpectraMax^®^ reader (Molecular Devices, Sunnyvale, CA USA). Methanol was used in the place of DPPH for the blank. Analyses were conducted in triplicate and the follow equation (*Eq.1*) was used to calculate the % of DPPH quenching (or percentage antioxidant activity).

% DPPH= {1 − [(Abs _sample_ – Abs _blank_)/Abs _negative control_]} (1)

The Hydroxyl Radical-Scavenging (HRS) assay was conducted according to the method that was described in Smirnoff and Cumbes [70], with some modifications [14]. Briefly, different concentrations of the extracts reacted with FeSO4 (1.5 mM), hydrogen peroxide (6 mM), and sodium salicylate (20 mM), at 37 °C for 1 h. After, the absorbance was measured at 562 nm while using a SpectraMax^®^ reader. 

EC_50_ values (concentration correspondent to 50% of the activity, in μg mL^−1^) were then calculated by plotting %DPPH or %OH scavenging against the concentrations of the samples.

### 3.4. Statistical Analysis and Correlation with Meteorological Data

The results of the content of different polyphenol classes (CA, A, K, L, M, Q, T), of TFC, TPC, and of EC_50_ of the leaf extracts were expressed as mean ± standard deviation (SD) (*n* = 3). A two-way ANOVA (factors: time of the day and month) followed by Tukey *post hoc* test were used to compare the different samples. A one-way ANOVA was used for the antioxidant capacity assays. The homogeneity of variance by Levene’s test and the normality of the data by Shapiro-Wilk test were evaluated. When necessary, a non-parametric ANOVA (in ranks) was applied.

To verify the possible correlation between the meteorological parameters (temperature and irradiance) and the content of the diverse polyphenols, a Person’s correlation coefficient (*r*) was calculated, with a correlation being considered as strong when *r* > 0.7, and as moderate when 0.3 < *r* < 0.7.

All of the statistical analyses were performed while using SigmaPlot^®^Systat^®^ software (version 12.5) and the differences were considered to be significant when *p* ≤ 0.05.

## 4. Conclusions

In our study, July at 13:00 was found to be the best harvesting moment for all of the polyphenolic classes of *P. latifolia*, for quercetin and tannin derivatives in *C. incanus* and only for tannins in *P. lentiscus*. The correlation with environmental parameters showed that they positively affected the polyphenol content of *C. incanus* and *P. latifolia*, while the antioxidant activity of the extracts only varied in this last species, with the greatest activity being obtained for July. Our results provide important information for determining the best harvesting moment (balsamic time) for *P. latifolia*, *C. incanus* and *P. lentiscus*. Moreover, the correlation between polyphenol content and meteorological data allows for understanding how each species responds to the surrounding environment. This can be important in the cultivation of these plants under controlled conditions to modulate their leaf phytochemical profile and increase the accumulation of their most valuable constituents.

## Figures and Tables

**Table 1:** Correlation (Pearson coefficient—*r*) between phenolic composition and environmental data (temperature and solar irradiance) for the three studied species (*Phillyrea latifolia*, *Cistus incanus* and *Pistacia lentiscus*).

Phenolic Composition	Temperature	Solar Irradiance
***P. latifolia***		
**K**	0.26	0.10
**A**	0.12	0.20
**CA**	0.54*	0.35
**Q**	0.52*	0.05
**L**	0.65**	0.14
**TFC**	0.59**	0.11
**TPC**	0.59**	0.12
***C. incanus***		
**K**	-0.28	-0.17
**M**	-0.02	0.13
**Q**	0.46*	0.67**
**T**	0.45*	0.49*
**TFC**	0.12	0.32
**TPC**	0.43	0.51*
***P. lentiscus***		
**M**	0.18	-0.07
**Q**	0.09	0.45
**T**	0.17	0.25
**TFC**	0.17	0.05
**TPC**	0.19	0.21

A—apigenin derivatives; CA—caffeic acid derivatives; K—kaempferol derivatives; L—luteolin derivatives; M—myricetin derivatives; Q—quercetin derivatives; T—tannin derivatives; TFC—total flavonoid content; and, TPC—total phenolic content. * and ** are significant at *p* ≤ 0.05 and 0.01 respectively.

**Table 2:** Antioxidant capacity (EC_50_ values, in μg mL^−1^) of *Phillyrea latifolia*, *Cistus incanus* and *Pistacia lentiscus* extracts from leaves harvested at 13.00, during the three different months (May, July and October), using 2,2-diphenyl-1-picrylhydrazyl (DPPH) and Hydroxyl Radical-Scavenging (HRS) assays.

	May		July		October	
	DPPH	HRS	DPPH	HRS	DPPH	HRS
***P. latifolia***	9.6 ± 1.0 ^a^	0.47 ± 0.05 ^a^	2.2 ± 0.4 ^b^	0.12 ± 0.02 ^b^	1.2 ± 0.2 ^c^	0.23 ± 0.03 ^c^
***C. incanus***	0.74 ± 0.12 ^a^	0.22 ± 0.03 ^a^	0.53 ± 0.08 ^a^	0.24 ± 0.04 ^a^	0.53 ± 0.05 ^a^	0.24 ± 0.03 ^a^
***P. lentiscus***	2.7 ± 0.4 ^a^	0.85 ± 0.11 ^a^	2.8 ± 0.3 ^a^	0.82 ± 0.13 ^a^	2.8 ± 0.4 ^a^	0.81 ± 0.10 ^a^

Mean ± SD (*n* = 3). Equal letters indicate no significant differences between the results for each species using the same method (*p* < 0.05).

**Table 3:** Details of the phenological stages of the three studied species (*Cistus incanus*, *Phillyrea latifolia* and *Pistacia lentiscus*) and of the meteorological parameters during the harvesting period.

Month and Year of Harvesting (season)	Phenological Stage			Meteorological Conditions		
	*Cistus incanus*	*Phillyrea latifolia*	*Pistacia lentiscus*	Monthly Cumulative Rainfall (mm)	Daily Temperature—Mean Values (°C)	Daily Global Solar Irradiance—Mean Values (W m^-2^)
**2014**						
***May (Spring)***	Blooming	Blooming	Blooming	48.0	17.8 ± 3.0	919.4 ± 75.6
***July (Summer)***	Fructification (End)	Fructification (Beginning)	Fructification (Beginning)	84.0	24.2 ± 2.8	847.1 ± 73.2
***October (Autumn)***	Vegetative	Fructification (End)	Fructification	173.8	19.3 ± 3.6	611.4 ± 91.7
**2015**						
***May (Spring)***	Blooming	Blooming	Blooming	53.4	20.8 ± 3.6	803.8 ± 146.6
***July (Summer)***	Fructification (End)	Fructification (Beginning)	Fructification (Beginning)	29.2	27.3 ± 2.9	857.2 ± 50.9
***October (Autumn)***	Vegetative	Fructification (End)	Fructification	70.6	20.9 ± 2.0	562.7 ± 118.1

## Supplementary Materials

The following are available online at https://www.mdpi.com/1420-3049/25/4/956/s1, Table S1: Daily and monthly phenolic composition of leaf ethanolic extracts of *Phillyrea latifolia* in two consecutive years (2014-2015); Table S2: Daily and monthly phenolic composition of leaf ethanolic extracts of *Cistus incanus* in two consecutive years (2014-2015); Table S3: Daily and monthly phenolic composition of leaf ethanolic extracts of *Pistacia lentiscus* in two consecutive years (2014-2015).
