# Supplementary material for: Seasonal and Diurnal Variation in Leaf Phenolics of Three Medicinal Mediterranean Wild Species: What Is the Best Harvesting Moment to Obtain the Richest and the Most Antioxidant Extracts?
Source: Molecules. 2020 Feb 20;25(4):956. doi: 10.3390/molecules25040956 (PMC7071013; doi:10.3390/molecules25040956)
Supplement: Supplementary file 1 [file molecules-25-00956-s001.pdf]

**Table S1.** Daily and monthly phenolic composition of leaf ethanolic extracts of *Phillyrea latifolia* in two consecutive years (2014–2015). A—apigenin derivatives; CA—caffeic acid derivatives; K—kaempferol derivatives; L—luteolin derivatives; Q—quercetin derivatives.

| *P. latifolia* | May | | | July | | | October | | |
|---|---|---|---|---|---|---|---|---|---|
| **2014** | *8.00* | *13.00* | *18.00* | *8.00* | *13.00* | *18.00* | *8.00* | *13.00* | *18.00* |
| K | $1.0 \pm 0.004^{aB}$ | $1.16 \pm 0.12^{aB}$ | $0.95 \pm 0.18^{aC}$ | $1.38 \pm 0.19^{bA}$ | $1.79 \pm 0.07^{aA}$ | $1.75 \pm 0.05^{aA}$ | $1.39 \pm 0.12^{bA}$ | $1.66 \pm 0.07^{aA}$ | $1.37 \pm 0.08^{bB}$ |
| A | $0.15 \pm 0.02^{aC}$ | $0.18 + 0.02^{aC}$ | $0.13 \pm 0.02^{aB}$ | $0.21 \pm 0.02^{bB}$ | $0.33 \pm 0.06^{aB}$ | $0.25 \pm 0.03^{bA}$ | $0.33 \pm 0.03^{bA}$ | $0.42 \pm 0.01^{aA}$ | $0.23 \pm 0.02^{cA}$ |
| CA | $0.57 \pm 0.02^{aC}$ | $0.73 \pm 0.02^{aC}$ | $0.44 \pm 0.03^{aB}$ | $0.92 \pm 0.13^{cB}$ | $3.04 \pm 0.28^{aA}$ | $1.31 \pm 0.21^{bA}$ | $1.30 \pm 0.17^{aA}$ | $1.61 \pm 0.27^{aB}$ | $1.33 \pm 0.21^{aA}$ |
| Q | $3.03 \pm 0.84^{bB}$ | $5.49 \pm 0.93^{aC}$ | $1.96 \pm 0.07^{bB}$ | $16.09 \pm 1.35^{bA}$ | $23.84 \pm 2.04^{aA}$ | $17.94 \pm 0.88^{bA}$ | $16.88 \pm 0.32^{bA}$ | $19.40 \pm 0.51^{aB}$ | $19.45 \pm 0.57^{aA}$ |
| L | $6.18 \pm 1.12^{aC}$ | $9.16 \pm 0.70^{aC}$ | $6.63 \pm 0.57^{aC}$ | $22.87 \pm 0.97^{bA}$ | $32.68 \pm 2.19^{aA}$ | $22.19 \pm 1.53^{bA}$ | $17.27 \pm 1.21^{bB}$ | $22.31 \pm 3.42^{aB}$ | $17.61 \pm 1.15^{bB}$ |
| **2015** | | | | | | | | | |
| K | $0.09 \pm 0.01^{aB}$ | $0.010 \pm 0.02^{aB}$ | $0.07 \pm 0.02^{aB}$ | $1.28 \pm 0.17^{aA}$ | $1.47 \pm 0.30^{aA}$ | $1.08 \pm 0.35^{aA}$ | $1.10 \pm 0.12^{aA}$ | $1.37 \pm 0.07^{aA}$ | $1.08 \pm 0.08^{aA}$ |
| A | $0.07 \pm 0.01^{aC}$ | $0.10 \pm 0.01^{aC}$ | $0.07 \pm 0.001^{aB}$ | $0.17 \pm 0.02^{bB}$ | $0.24 \pm 0.03^{aB}$ | $0.18 \pm 0.01^{bA}$ | $0.27 \pm 0.03^{bA}$ | $0.37 \pm 0.05^{aA}$ | $0.20 \pm 0.02^{cA}$ |
| CA | $0.34 \pm 0.02^{abB}$ | $0.52 \pm 0.03^{aC}$ | $0.22 \pm 0.02^{bC}$ | $0.93 \pm 0.08^{cA}$ | $2.15 \pm 0.27^{aA}$ | $1.32 \pm 0.11^{bA}$ | $0.91 \pm 0.15^{bA}$ | $1.32 \pm 0.11^{aB}$ | $1.03 \pm 0.15^{bB}$ |
| Q | $2.25 \pm 0.20^{bC}$ | $4.70 \pm 0.52^{aC}$ | $1.63 \pm 0.10^{bC}$ | $16.08 \pm 0.12^{cA}$ | $21.86 \pm 0.41^{aA}$ | $17.92 \pm 1.39^{bA}$ | $13.89 \pm 0.92^{bB}$ | $16.77 \pm 0.85^{aB}$ | $16.16 \pm 1.34^{aB}$ |
| L | $4.89 \pm 0.15^{bC}$ | $8.42 \pm 0.64^{aC}$ | $5.83 \pm 0.90^{bC}$ | $19.28 \pm 0.36^{bA}$ | $20.69 \pm 0.98^{bA}$ | $25.92 \pm 1.87^{aA}$ | $15.13 \pm 1.22^{aB}$ | $16.49 \pm 1.76^{aB}$ | $12.86 \pm 0.33^{bB}$ |

Mean values ± SD in μmol $gDW^{-1}$ ($n = 3$). The data were analyzed by two-way ANOVA test, with Tukey post-test, after variance homogeneity analysis by Levene's test and normality analysis by Shapiro-Wilk test. When necessary, a non-parametric ANOVA (in ranks) was applied. Equal capital letters indicate no statistical differences between the monthly results (at the same time) and lowercase letters indicate no statistical differences between daily results (inside the same month), with $p \leq 0.05$.

**Table S2.** Daily and monthly phenolic composition of leaf ethanolic extracts of *Cistus incanus* in two consecutive years (2014–2015). K—kaempferol derivatives; M—myricetin derivatives; Q—quercetin derivatives; T—tannin derivatives.

| *C.incanus* | **May** | | | **July** | | | **October** | | |
|---|---|---|---|---|---|---|---|---|---|
| **2014** | *8.00* | *13.00* | *18.00* | *8.00* | *13.00* | *18.00* | *8.00* | *13.00* | *18.00* |
| K | 0.34 ± 0.04$^{bB}$ | 0.45 ± 0.04$^{aB}$ | 0.44 ± 0.03$^{bB}$ | 0.37 ± 0.11$^{bB}$ | 0.63 ± 0.21$^{aB}$ | 0.42 ± 0.03$^{bB}$ | 0.79 ± 0.08$^{bA}$ | 1.02 ± 0.21$^{aA}$ | 0.94 ± 0.12$^{bA}$ |
| M | 12.56 ± 0.35$^{cB}$ | 18.85 ± 3.57$^{bA}$ | 24.71 ± 0.48$^{aA}$ | 16.24 ± 0.98$^{aA}$ | 15.08 ± 0.82$^{aA}$ | 13.52 ± 0.81$^{aB}$ | 11.95 ± 0.24$^{aB}$ | 12.89 ± 0.43$^{aB}$ | 12.07 ± 0.49$^{aB}$ |
| Q | 4.18 ± 0.23$^{bB}$ | 5.78 ± 0.17$^{aB}$ | 4.17 ± 0.14$^{bB}$ | 6.36 ± 0.28$^{bA}$ | 9.73 ± 1.16$^{aA}$ | 6.10 ± 1.23$^{bA}$ | 5.83 ± 0.94$^{aA}$ | 7.00 ± 0.32$^{aB}$ | 4.06 ± 0.19$^{bB}$ |
| T | 21.43 ± 3.02$^{cB}$ | 45.02 ± 8.55$^{aA}$ | 33.14 ± 2.00$^{bAB}$ | 37.55 ± 8.58$^{aA}$ | 34.26 ± 7.75$^{aAB}$ | 43.70 ± 1.75$^{aA}$ | 28.31 ± 3.21$^{aAB}$ | 23.93 ± 4.94$^{aB}$ | 29.60 ± 4.86$^{aB}$ |
| **2015** | | | | | | | | | |
| K | 0.32 ± 0.04$^{bB}$ | 0.44 ± 0.04$^{aB}$ | 0.43 ± 0.03$^{bB}$ | 0.35 ± 0.03$^{aB}$ | 0.46 ± 0.09$^{aB}$ | 0.37 ± 0.06$^{aB}$ | 0.77 ± 0.08$^{aA}$ | 0.79 ± 0.15$^{aA}$ | 0.92 ± 0.12$^{aA}$ |
| M | 12.49 ± 0.34$^{cB}$ | 21.31 ± 2.19$^{bA}$ | 24.65 ± 0.48$^{aA}$ | 15.13 ± 0.90$^{aA}$ | 14.69 ± 0.57$^{aB}$ | 12.27 ± 1.33$^{bB}$ | 11.57 ± 0.09$^{aB}$ | 12.62 ± 0.38$^{aC}$ | 11.93 ± 0.94$^{aB}$ |
| Q | 5.04 ± 0.40$^{aA}$ | 6.87 ± 1.09$^{aA}$ | 4.97 ± 0.10$^{bA}$ | 5.53 ± 0.78$^{aA}$ | 6.03 ± 0.21$^{aA}$ | 4.26 ± 0.25$^{bA}$ | 5.35 ± 1.10$^{aA}$ | 6.15 ± 0.27$^{aA}$ | 3.78 ± 0.22$^{bA}$ |
| T | 21.43 ± 3.02$^{cA}$ | 53.95 ± 0.17$^{aB}$ | 33.14 ± 2.00$^{bA}$ | 26.19 ± 1.35$^{bA}$ | 86.92 ± 7.42$^{aA}$ | 19.73 ± 7.77$^{bB}$ | 22.79 ± 1.54$^{aA}$ | 21.27 ± 2.92$^{aC}$ | 18.27 ± 3.23$^{aB}$ |

Mean values ± SD in μmol $gDW^{-1}$ ($n = 3$). The data were analyzed by two-way ANOVA test, with Tukey post-test, after variance homogeneity analysis by Levene's and normality analysis by Shapiro-Wilk test. When necessary, a non-parametric ANOVA (in ranks) was applied. Equal capital letters indicate no statistical differences between the monthly results (at the same time) and lowercase letters indicate no statistical differences between daily results (inside the same month), with $p \leq 0.05$.

**Table S3.** Daily and monthly phenolic composition of leaf ethanolic extracts of *Pistacia lentiscus* in two consecutive years (2014–2015). M—myricetin derivatives; Q—quercetin derivatives; T—tannin derivatives.

| *P.lentiscus* | **May** | | | **July** | | | **October** | | |
|---|---|---|---|---|---|---|---|---|---|
| **2014** | *8.00* | *13.00* | *18.00* | *8.00* | *13.00* | *18.00* | *8.00* | *13.00* | *18.00* |
| M | 17.46 ± 1.99$^{aC}$ | 13.41 ± 0.82$^{bC}$ | 13.28 ± 0.42$^{bC}$ | 22.50 ± 1.12$^{aA}$ | 20.21 ± 1.27$^{aA}$ | 21.21 ± 0.46$^{aA}$ | 20.09 ± 0.98$^{aB}$ | 18.89 ± 1.48$^{bB}$ | 17.59 ± 1.92$^{bB}$ |
| Q | 4.85 ± 0.51$^{aA}$ | 5.15 ± 0.40$^{aB}$ | 4.34 ± 0.67$^{aA}$ | 4.75 ± 0.29$^{bA}$ | 6.91 ± 0.46$^{aA}$ | 5.41 ± 1.38$^{aA}$ | 5.36 ± 0.11$^{aA}$ | 5.74 ± 0.24$^{aB}$ | 4.41 ± 0.06$^{bA}$ |
| T | 51.26 ± 1.89$^{bB}$ | 48.34 ± 1.73$^{bC}$ | 55.21 ± 2.56$^{aA}$ | 74.87 ± 1.20$^{bA}$ | 79.84 ± 4.77$^{aA}$ | 47.47 ± 2.11$^{cB}$ | 51.52 ± 0.10$^{bB}$ | 56.70 ± 0.20$^{aB}$ | 56.71 ± 2.56$^{aA}$ |
| **2015** | | | | | | | | | |
| M | 12.72 ± 1.83$^{aB}$ | 10.27 ± 1.75$^{aB}$ | 10.26 ± 1.03$^{aC}$ | 12.14 ± 0.80$^{bB}$ | 18.65 ± 0.58$^{aA}$ | 19.32 ± 3.66$^{aA}$ | 17.48 ± 0.85$^{aA}$ | 16.44 ± 1.29$^{aA}$ | 15.41 ± 1.73$^{aB}$ |
| Q | 3.41 ± 0.26$^{aB}$ | 3.80 ± 0.25$^{aC}$ | 3.13 ± 0.38$^{aA}$ | 2.69 ± 0.29$^{bB}$ | 5.70 ± 0.17$^{aA}$ | 3.41 ± 0.25$^{bA}$ | 4.36 ± 0.28$^{aA}$ | 4.66 ± 0.54$^{aB}$ | 3.51 ± 0.62$^{aA}$ |
| T | 35.00 ± 2.15$^{bB}$ | 43.75 ± 4.38$^{aA}$ | 40.62 ± 2.91$^{abAB}$ | 55.80 ± 0.46$^{aA}$ | 42.38 ± 1.09$^{bA}$ | 38.83 ± 2.85$^{bB}$ | 38.75 ± 4.00$^{bB}$ | 45.00 ± 2.52$^{abA}$ | 45.67 ± 4.19$^{aA}$ |

Mean values ± SD in µmol $gDW^{-1}$ ($n = 3$). The data were analyzed by two-way ANOVA test, with Tukey post-test, after variance homogeneity analysis by Levene's and normality analysis by Shapiro-Wilk test. When necessary, a non-parametric ANOVA (in ranks) was applied. Equal capital letters indicate no statistical differences between the monthly results (at the same time) and lowercase letters indicate no statistical differences between daily results (inside the same month), with $p \leq 0.05$.
